# Cardiac Tamponade—A Rare Manifestation of Ruxolitinib Discontinuation Syndrome: A Case Report

**DOI:** 10.1155/cric/8854255

**Published:** 2026-05-04

**Authors:** Shariq Hamid, Rory Bennet, Jessica Yao, Joshua Wong

**Affiliations:** ^1^ Department of General Medicine, Royal Melbourne Hospital, Parkville, Victoria, Australia, mh.org.au; ^2^ Department of Haematology, Royal Melbourne Hospital, Parkville, Victoria, Australia, mh.org.au; ^3^ Department of Haematology, Peter MacCallum Cancer Centre, Parkville, Victoria, Australia, petermac.org; ^4^ Department of Cardiology, Royal Melbourne Hospital, Parkville, Victoria, Australia, mh.org.au; ^5^ Melbourne Medical School, University of Melbourne, Melbourne, Victoria, Australia, unimelb.edu.au; ^6^ Department of Cardiology, Peter MacCallum Cancer Centre, Parkville, Victoria, Australia, petermac.org

## Abstract

A 52‐year‐old gentleman presenting with tachycardia was found to have a large pericardial effusion and cardiac tamponade. This case was on a background of long‐standing myelofibrosis managed with ruxolitinib, which was recently withheld for an orthopaedic procedure. He was diagnosed with presumed ruxolitinib discontinuation syndrome (RDS), resulting in a large pericardial effusion due to an increased inflammatory response. He was managed with a pericardial window and prompt recommencement of ruxolitinib and steroids. This case highlights the importance and increasing prevalence of RDS with the serious consequence of cardiac tamponade. Symptoms of RDS should be recognised and managed with recommencement of a JAK inhibitor and steroids.

## 1. Introduction

Ruxolitinib (RUX) is an inhibitor of Janus Kinase 1 and Janus Kinase 2 (JAK1 and JAK2). RUX is the standard treatment of certain patients with myelofibrosis [1], and it has also recently been approved for use in graft‐versus‐host disease. Multiple trials are currently underway to explore its use in rheumatology and dermatology, and it is expected that RUX use will increase, as multiple disease processes may benefit from the inhibition of JAK1. Sudden discontinuation of RUX can result in a systemic inflammatory response defined as ruxolitinib discontinuation syndrome (RDS). Here, we report a case of developing pericardial effusion and resultant cardiac tamponade requiring surgical intervention after sudden discontinuation of RUX.

## 2. Case Presentation

A 52‐year‐old gentleman presented to his scheduled day medical unit for a blood transfusion in the setting of long‐standing myelofibrosis. He was referred in for admission after being found to be persistently tachycardic, although he remained asymptomatic. He denied chest pain or dyspnoea; however, he was recovering from a recent left femoral shaft fracture, which was surgically managed with an intramedullary nail 3 weeks prior, during which time his RUX for his myelofibrosis had been withheld due to persistent anaemia and pancytopenia.

On examination, the patient was tachycardic at 125 bpm, oxygen saturations were 99% on room air, blood pressure was 130/70 mmHg and he was febrile at 39.5°C. The physical exam was unremarkable, but he was noted to have an active flare of his gout in his left ankle.

## 3. Past Medical History

The patient had an extensive past medical history, mostly secondary to myelofibrosis with a JAK2 V617F mutation diagnosed 11 years prior in 2012. As a result of this, he had splenomegaly, haemorrhagic complications including scrotal hematoma and thrombotic complications including DVT, PE and splenic infarcts. He was managed with RUX 15 mg twice daily (which had recently been abruptly ceased in the setting of the left midshaft femoral fracture) and was also managed with blood transfusions as required. An allogenic stem cell transplant had been considered but not offered due to the patient′s performance status.

The patient had commenced RUX for his myelofibrosis in 2021, and this had been up‐titrated to a dose of 15 mg twice daily. One year earlier, due to issues with pancytopenia and in the setting of a COVID‐19 infection, his RUX had been withheld for 4 months, with a tapering dose of steroids used in the interim. The patient did not have any adverse symptoms associated with RUX withdrawal.

His other past medical history included gout on allopurinol, Type 2 diabetes on insulin and chronic back pain.

## 4. Differential Diagnosis

The main differential diagnoses included sepsis in the setting of an immunocompromised host (from marrow dysfunction) and acute pulmonary embolism, given the patient′s previous thrombotic events and recent orthopaedic surgery. A computed tomography pulmonary angiogram (CTPA) excluded a pulmonary embolism but revealed the presence of a large circumferential pericardial effusion.

The aetiology of the pericardial effusion was then considered. The leading differential was a malignant effusion in the setting of possible conversion from myelofibrosis to acute myeloid leukaemia. Other causes included pericardial effusion due to hyperuricemic pericarditis in the setting of a high uric acid state from increased cell turnover and haemorrhagic pericardial effusion secondary to thrombocytopenia.

## 5. Investigations

Initial investigations included laboratory testing, blood and urine cultures and a chest x‐ray. The patient was notably bicytopenic (haemoglobin 81 g/L, platelets 78 × 10^9^/L) with an elevated white cell count (31 × 10^9^/L) and C‐reactive protein (CRP) 83.5 mg/L. Ferritin was markedly elevated at 2331 *μ*g/L, acknowledging a baseline elevation of 1200 *μ*g/L. Biochemistry revealed all electrolytes were within normal limits, but an acute kidney injury was noted (creatinine 1.8 mg/dL, from a baseline of 0.88 mg/dL).

**Figure 1:**
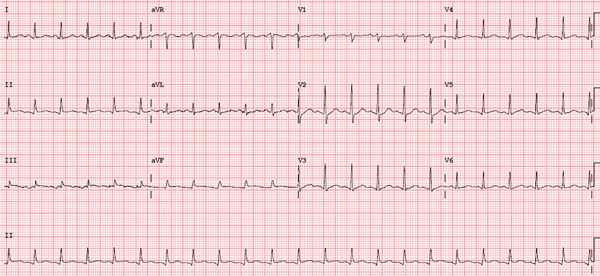
ECG demonstrating sinus tachycardia.

**Figure 2:**
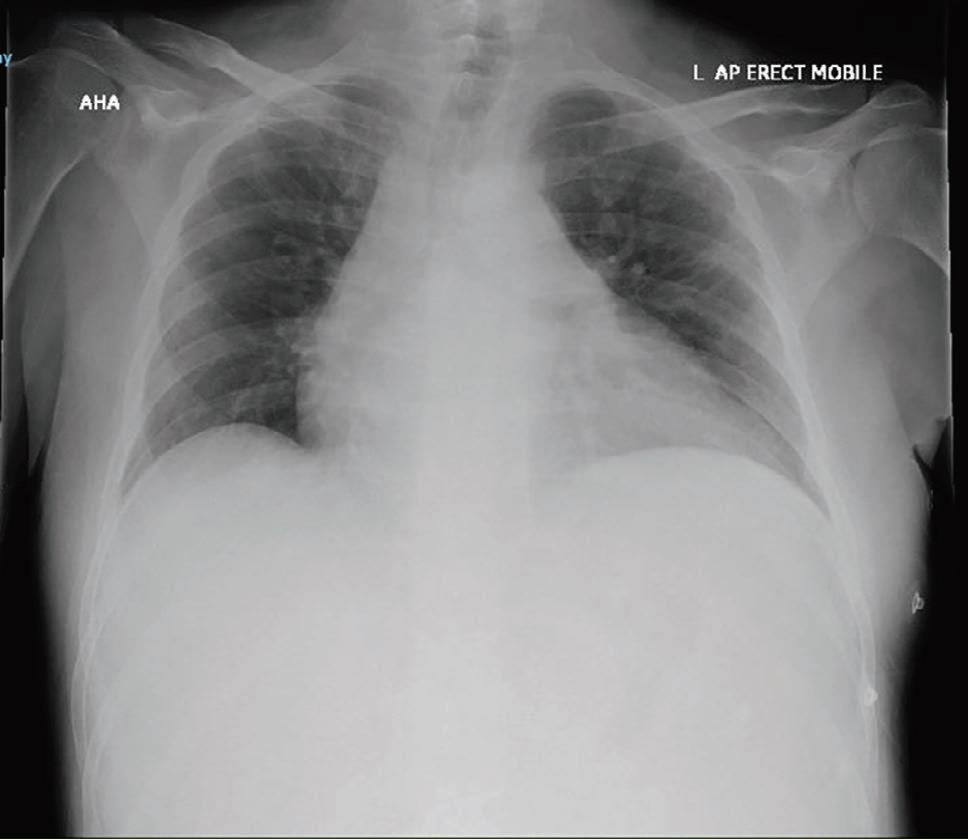
AP chest x‐ray demonstrating enlarged cardiac silhouette.

**Figure 3:**
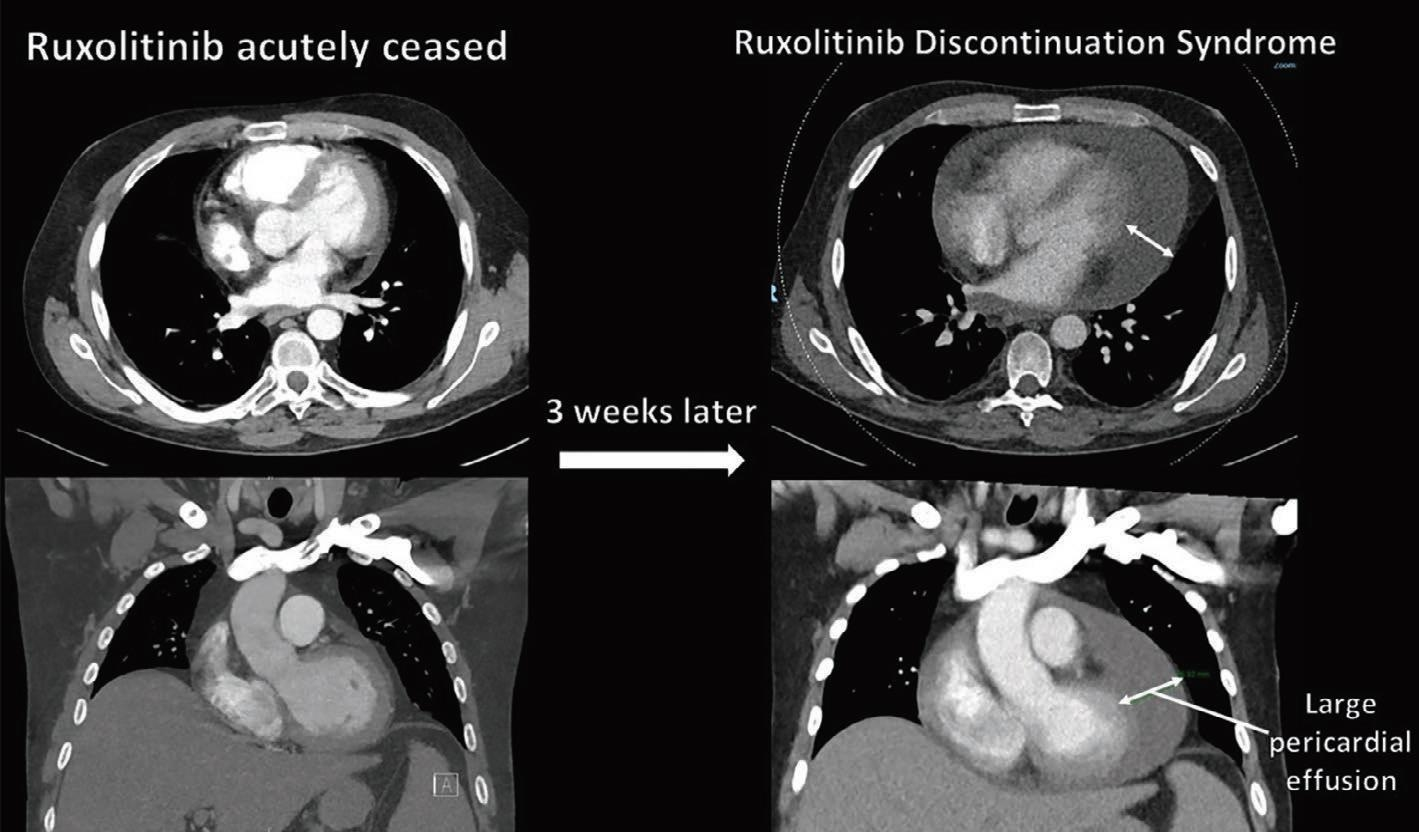
Serial computed tomography pulmonary angiogram (CTPA) showing rapid accumulation of pericardial effusion.

Electrocardiography (Figure 1) demonstrated sinus tachycardia with normal voltages and no evidence of pericarditis. A chest x‐ray demonstrated an enlarged cardiac silhouette (even accounting for AP projection) (Figure 2). CTPA demonstrated a large pericardial effusion measuring 37 mm in depth (Figure 3). This was compared to a CTPA from 3 weeks prior, which had previously demonstrated a trivial pericardial effusion measuring 7 mm, which was stable to previous imaging. This confirmed the rapid accumulation of pericardial fluid over the 3‐week period.

**Figure 4:**
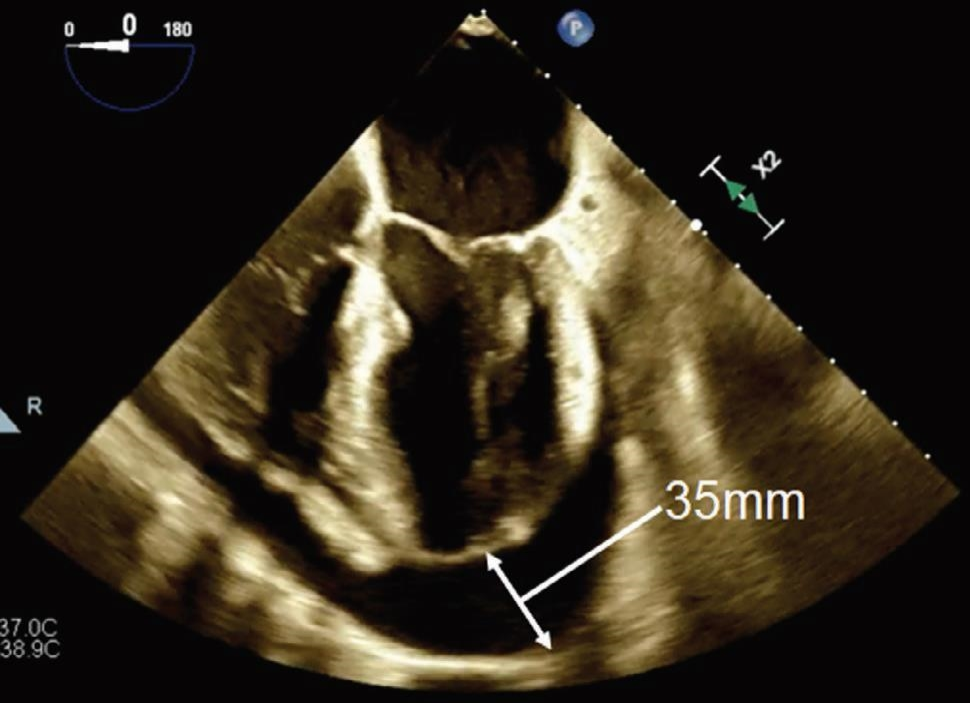
Transesophageal echocardiogram demonstrating a large pericardial effusion around the posterior left ventricle.

A subsequent transthoracic echocardiogram (TTE) and transesophageal echocardiogram (TOE) demonstrated a large pericardial effusion (loculated around the posterior LV wall) with echocardiographic features of tamponade, including right atrial chamber collapse, inspiratory inflow variation and inferior vena cava plethora (Figure 4).

A repeat clinical exam confirmed the presence of pulsus paradoxus.

## 6. Management

An urgent pericardial window and drainage via left thoracotomy were performed. The posterior location of effusion and significant thrombocytopenia increased the risk of bleeding with percutaneous drainage, and so this was not attempted. Eight hundred millilitres of serous fluid was drained. Culture was negative with no malignant infiltrate. There was a high predominance of neutrophils, which is thought to be inflammatory in nature.

Concurrently, broad‐spectrum antibiotics (piperacillin/tazobactam) were commenced despite a negative septic screen. The left knee swelling was aspirated, revealing urate crystals and confirming gout; colchicine was commenced.

Given the ongoing fevers, tachycardia, pericardial effusion and acute gout flare, a systemic inflammatory process was thought to be occurring. RUX compliance history was taken, and an abrupt cessation 3 weeks ago was confirmed with no tapering or steroid crossover. This temporal association of RUX cessation with the development of a large pericardial effusion and inflammatory process was thought to be consistent with RDS. This is a systemic inflammatory response due to cytokine release. The patient was promptly recommenced on RUX and high‐dose steroids (1 mg/kg).

The patient improved clinically after drainage and remained afebrile once RUX was recommenced. He was discharged home 5 days postoperatively.

## 7. Discussion

**Figure 5:**
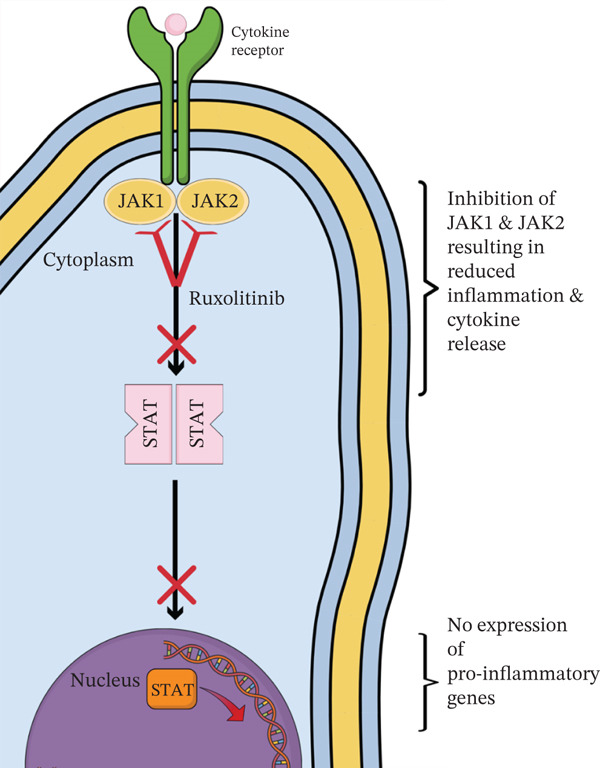
Mechanism of action of ruxolitinib.

**Figure 6:**
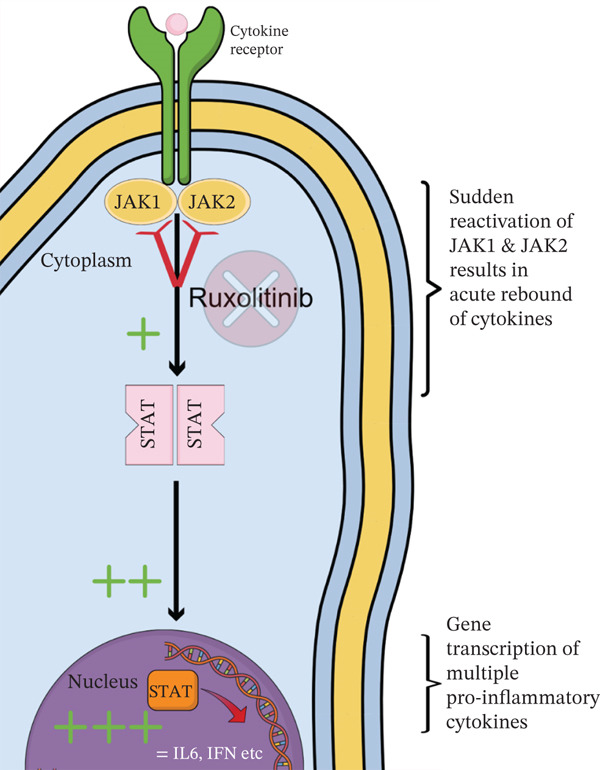
Pathophysiology of ruxolitinib discontinuation syndrome.

JAK inhibitors such as RUX work by suppressing downstream signalling pathways of inflammatory cytokines (Figure 5) [2]. Due to its adverse effects, specifically the myelosuppression from JAK2 inhibition, between 51% [3] and 89% [4] of patients discontinue RUX within 3 years of therapy. In early Phase 1 and 2 studies of RUX in myelofibrosis, most patients experienced relapse of their symptoms and worsening splenomegaly after RUX discontinuation [5]. In a small proportion, an acute rebound of inflammatory cytokines caused severe symptoms such as acute respiratory distress syndrome, systemic inflammatory response syndrome and DIC‐like syndrome, which was defined as severe RDS (Figure 6) [4].

Severe RDS is still relatively uncommon, and only a few cases have been reported. Pericardial effusion has been reported before as a feature of RDS [6, 7]; however, to our knowledge, this case represents the first report of cardiac tamponade due to the effusion. RDS is hypothesised to occur due to the sudden reactivation of JAK1/JAK2, causing a cytokine storm and systemic inflammatory response as JAK1 triggers the production of multiple proinflammatory cytokines such as IL‐6 and IFN [8]. RDS has been recognised to occur between 48 h and 3 weeks of cessation of RUX, which fits in the timeline of our case. Due to the risk of RDS, guidelines currently recommend tapering RUX and using steroids if there is any sign of RDS [9]. There is a paucity in the evidence regarding treating RDS aside from treatment of complications, steroids and prompt reintroduction of RUX or possibly a second‐generation JAK inhibitor. Symptoms such as fever and ARDS usually resolve within 48 h of reintroducing RUX [10].

## 8. Follow‐Up

After RUX recommencement, our patient experienced resolution of fevers and serial reduction in CRP levels. Follow‐up TTE 3 weeks later demonstrated no reaccumulation of his effusion.

Unfortunately, this patient passed away 2 months postdischarge with features suspected of leukaemic transformation (new lymphadenopathy and progressive marrow failure). It is suspected that this disease biology also contributed to the inflammatory state seen at the time of RUX discontinuation. However, it is difficult to know to what degree the pericardial effusion can be attributed to disease progression; no malignant cells were detected in the aspirate, and after recommencement of RUX and steroids, the repeat TTE 2 months postdischarge was stable from discharge. We hypothesise his initial presentation was a combination of RDS and a heralding of disease progression.

## 9. Conclusion

Severe RDS is a relatively rare condition occurring days to weeks after the sudden cessation of JAK inhibitor therapy. It may present with acute respiratory distress syndrome, septic‐like shock and systemic inflammatory response syndrome. Pericardial effusion and cardiac tamponade are rare manifestations of this heightened inflammatory state.

As RUX prescription increases, it is important for clinicians to be aware of RDS as a rare cause for pericardial effusions. Early recognition and targeted treatment with recommencement of RUX and corticosteroids can result in rapid clinical improvement.

### 9.1. Learning Points

1. RDS is a rare condition occurring days to weeks after sudden RUX cessation, which can present as acute respiratory distress syndrome, septic‐like shock and systemic inflammatory response syndrome.
2. Pericardial effusion and cardiac tamponade are rare inflammatory manifestations of severe RDS.
3. Tapering of RUX reduces the risk of RDS.
4. Early recognition of RDS and targeted treatment with recommencement of RUX and corticosteroids can result in rapid clinical improvement.

## Funding

Open access publishing is facilitated by the University of Melbourne, as part of the Wiley–The University of Melbourne agreement via the Council of Australasian University Librarians.

## Disclosure

All authors have read and approved the final version of the manuscript. Dr Joshua Wong had full access to all of the data in this study and takes complete responsibility for the integrity of the data and the accuracy of the data analysis. Dr Shariq Hamid affirms that this manuscript is an honest, accurate and transparent account of the study being reported; that no important aspects of the study have been omitted and that any discrepancies from the study as planned have been explained. This case was presented as an abstract in both the 72^nd^ Annual Scientific Meeting of the Cardiac Society of Australia and New Zealand, 1–4 August 2024, Perth, Western Australia, and in the European Society of Cardiology Congress 2024, 30 August–2 September, London, England.

## Ethics Statement

This article does not contain any studies with human or animal subjects performed by any of the authors. Informed patient consent was attained regarding the publication of this report.

## Conflicts of Interest

The authors declare no conflicts of interest.

## Data Availability

Data sharing is not applicable to this article as no datasets were generated or analysed during the current study.

## References

[1] Passamonti F. and Mora B. , Myelofibrosis, Blood. (2023) 141, no. 16, 1954–1970, 10.1182/blood.2022017423, 36416738. 36416738 PMC10646775

[2] Tanaka Y. , Luo Y. , O’Shea J. J. , and Nakayamada S. , Janus Kinase-Targeting Therapies in Rheumatology: A Mechanisms-Based Approach, Nature Reviews Rheumatology. (2022) 18, no. 3, 133–145, 10.1038/s41584-021-00726-8, 34987201. 34987201 PMC8730299

[3] Palandri F. , Breccia M. , Bonifacio M. , Polverelli N. , Elli E. M. , Benevolo G. , Tiribelli M. , Abruzzese E. , Iurlo A. , Heidel F. H. , Bergamaschi M. , Tieghi A. , Crugnola M. , Cavazzini F. , Binotto G. , Isidori A. , Sgherza N. , Bosi C. , Martino B. , Latagliata R. , Auteri G. , Scaffidi L. , Griguolo D. , Trawinska M. , Cattaneo D. , Catani L. , Krampera M. , Lemoli R. M. , Cuneo A. , Semenzato G. , Foà R. , di Raimondo F. , Bartoletti D. , Cavo M. , Palumbo G. A. , and Vianelli N. , Life After Ruxolitinib: Reasons for Discontinuation, Impact of Disease Phase, and Outcomes in 218 Patients With Myelofibrosis, Cancer. (2020) 126, no. 6, 1243–1252, 10.1002/cncr.32664, 31860137. 31860137

[4] Tefferi A. and Pardanani A. , Serious Adverse Events During Ruxolitinib Treatment Discontinuation in Patients With Myelofibrosis, Mayo Clinic Proceedings. (2011) 86, no. 12, 1188–1191, 10.4065/mcp.2011.0518, 2-s2.0-83155182714, 22034658. 22034658 PMC3228619

[5] Verstovsek S. , Kantarjian H. , Mesa R. A. , Pardanani A. D. , Cortes-Franco J. , Thomas D. A. , Estrov Z. , Fridman J. S. , Bradley E. C. , Erickson-Viitanen S. , Vaddi K. , Levy R. , and Tefferi A. , Safety and Efficacy of INCB018424, a JAK1 and JAK2 Inhibitor, in Myelofibrosis, New England Journal of Medicine. (2010) 363, no. 12, 1117–1127, 10.1056/NEJMoa1002028, 2-s2.0-77956696835, 20843246. 20843246 PMC5187954

[6] Beauverd Y. and Samii K. , Acute Respiratory Distress Syndrome in a Patient With Primary Myelofibrosis After Ruxolitinib Treatment Discontinuation, International Journal of Hematology. (2014) 100, no. 5, 498–501, 10.1007/s12185-014-1628-5, 2-s2.0-84936743553, 25034748. 25034748 PMC7100122

[7] Coltro G. , Mannelli F. , Guglielmelli P. , Pacilli A. , Bosi A. , and Vannucchi A. M. , A Life-Threatening Ruxolitinib Discontinuation Syndrome, American Journal of Hematology. (2017) 92, no. 8, 833–838, 10.1002/ajh.24775, 2-s2.0-85020117788, 28457008. 28457008

[8] Spinelli F. R. , Colbert R. A. , and Gadina M. , JAK1: Number One in the Family; Number One in Inflammation?, Rheumatology.(2021) 60, no. supplement_2, 10.1093/rheumatology/keab024, 33950229. PMC8599761 33950229

[9] Mascarenhas J. , Nguyen H. , Saunders A. , Oliver L. , Tomkinson H. , Perry R. , and McBride A. , Defining Ruxolitinib Failure and Transition to Next-Line Therapy for Patients With Myelofibrosis: A Modified Delphi Panel Consensus Study, Future Oncology. (2023) 19, no. 11, 763–773, 10.2217/fon-2022-1298, 37161798. 37161798

[10] Palandri F. , Palumbo G. A. , Elli E. M. , Polverelli N. , Benevolo G. , Martino B. , Abruzzese E. , Tiribelli M. , Tieghi A. , Latagliata R. , Cavazzini F. , Bergamaschi M. , Binotto G. , Crugnola M. , Isidori A. , Caocci G. , Heidel F. , Pugliese N. , Bosi C. , Bartoletti D. , Auteri G. , Cattaneo D. , Scaffidi L. , Trawinska M. M. , Stella R. , Ciantia F. , Pane F. , Cuneo A. , Krampera M. , Semenzato G. , Lemoli R. M. , Iurlo A. , Vianelli N. , Cavo M. , Breccia M. , and Bonifacio M. , Ruxolitinib Discontinuation Syndrome: Incidence, Risk Factors, and Management in 251 Patients With Myelofibrosis, Blood Cancer Journal. (2021) 11, no. 1, 10.1038/s41408-020-00392-1, 33414394. PMC7791065 33414394

